# Negative regulation of NF-κB signaling in T lymphocytes by the ubiquitin-specific protease USP34

**DOI:** 10.1186/1478-811X-11-25

**Published:** 2013-04-16

**Authors:** Konstantinos Poalas, Emeline M Hatchi, Nelia Cordeiro, Sonia M Dubois, Héloïse M Leclair, Claire Leveau, Catherine Alexia, Julie Gavard, Aimé Vazquez, Nicolas Bidère

**Affiliations:** 1 INSERM UMR_S 1014, Hôpital Paul Brousse, Villejuif 94800, France; 2 Université Paris-Sud P11, Orsay 91400, France; 3 Equipe Labellisée Ligue contre le Cancer, Villejuif, France; 4 Université Paris Descartes, Sorbonne Paris Cite, 6 rue de l’Ecole de Medecine, Paris 75006, France; 5 INSERM, U1016, 22 rue Méchain, Paris 75014, France; 6 CNRS, UMR8104, 22 rue Méchain, Paris 75014, France

**Keywords:** DUBs, NF-κB, Ubiquitinylation, T-Cell receptor

## Abstract

**Background:**

NF-κB is a master gene regulator involved in plethora of biological processes, including lymphocyte activation and proliferation. Reversible ubiquitinylation of key adaptors is required to convey the optimal activation of NF-κB. However the deubiquitinylases (DUBs), which catalyze the removal of these post-translational modifications and participate to reset the system to basal level following T-Cell receptor (TCR) engagement continue to be elucidated.

**Findings:**

Here, we performed an unbiased siRNA library screen targeting the DUBs encoded by the human genome to uncover new regulators of TCR-mediated NF-κB activation. We present evidence that knockdown of Ubiquitin-Specific Protease 34 (USP34) selectively enhanced NF-κB activation driven by TCR engagement, similarly to siRNA against the well-characterized DUB cylindromatosis (CYLD). From a molecular standpoint, USP34 silencing spared upstream signaling but led to a more pronounced degradation of the NF-κB inhibitor IκBα, and culminated with an increased DNA binding activity of the transcription factor.

**Conclusions:**

Collectively, our data unveils USP34 as a new player involved in the fine-tuning of NF-κB upon TCR stimulation.

## Findings

Nuclear factor-κB (NF-κB) transcription factors initiate transcription of genes essential for mounting an adequate immune response [1]. Ubiquitously expressed NF-κB heterodimers of Rel family proteins are normally sequestered in the cytosol of the cells by Inhibitors of NF-κB (IκBs) proteins [2]. In lymphocytes, the ligation of antigen receptors assembles the so-called CBM complex that consists of the scaffold CARMA1 and the heterodimer BCL10/MALT1 [3]. The CBM microenvironment drives oligomerized BCL10 and MALT1 to undergo K63-linked non-degradative ubiquitinylation [4-7]. This authorizes the recruitment and activation of the IκB kinase (IKK) complex that comprises two catalytic subunits (IKKα and IKKβ) and a regulatory subunit (NEMO, also called IKKγ) [8]. IKK phosphorylation of IκBs precipitates their K48-linked ubiquitinylation and proteasomal elimination, and thereby allows NF-κB to translocate to the nucleus where it binds DNA and initiates transcription [8]. NF-κB-dependent neosynthesis of IκBs subsequently drives NF-κB to shuttle back to the cytosol [1]. Although reversible ubiquitinylation processes are central for T-cell receptor-(TCR)-mediated NF-κB activation, the deubiquitinylases (DUBs) in charge of trimming these poly-ubiquitin chains to ensure optimal signaling, as well as to reset the system to basal levels remain poorly defined [9]. Thus far, two DUBs, namely cylindromatosis (CYLD) and A20 (also known as TNFAIP3), were demonstrated to negatively regulate antigen receptor signaling [9,10]. Herein, we provide evidence that Ubiquitin-Specific Protease 34 (USP34) also contributes to the fine-tuning of NF-κB upon TCR engagement.

**Figure 1:**
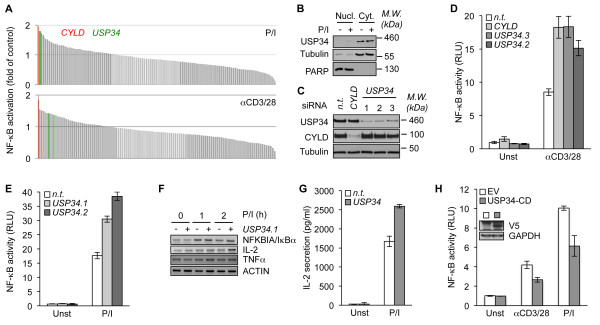
**Identification of USP34 as a negative regulator of TCR-mediated NF-κB activation. (A**) NF-κB reporter luciferase assay screen of a siRNA library targeting 98 DUBs (2 siRNA/target) in Jurkat T lymphocytes stimulated with 20 ng.ml^-1^ PMA plus 300 ng.ml^-1^ ionomycin (P/I, top panel), or with 0.5 μg.ml^-1^ anti-CD3 and anti-CD28 (bottom panel). Fold activation compared to non-targeting (n.t.) siRNA-treated cells is shown. Green and red histograms indicate siRNA against USP34 and CYLD, respectively. (**B**) Nuclear (Nucl.) and cytosolic (Cyt.) fractions from Jurkat T cells stimulated with P/I as in (**A**) for 0 and 15 min were analyzed by immunoblot. PARP and tubulin served as loading and purity controls for nucleus and cytosol, respectively. Molecular weight markers (*M.W.*) are indicated. (**C**) Lysates from Jurkat cells transfected for four days with siRNA against CYLD, USP34 (three individual sequences), or with control n.t. siRNA were analyzed by immunoblot as indicated. (**D and E**) NF-κB reporter luciferase assay (mean ± S.D. of triplicate experiments) of n.t.-, CYLD- or USP34-silenced Jurkat cells stimulated as in (A). RLU, Relative Light Units; Unst, unstimulated cells. (**F**) Cells as in (**C**) were stimulated with 20 ng.ml^-1^ PMA plus 300 ng.ml^-1^ ionomycin (P/I) for 1 and 2 hours. mRNA levels of NFKBIA (IκBα), IL-2, TNFα, and ACTIN were measured by RT-PCR. (**G**) Enzyme-Linked ImmunoSorbent Assay (ELISA) of IL-2 secreted in the supernatant of Jurkat treated as in (**C**) and stimulated with P/I. (**H**) NF-κB reporter luciferase assay of Jurkat cells transfected with the catalytic domain of USP34 (V5-tagged USP34-CD) or with a control empty vector (EV) and stimulated as indicated. Histograms represent mean ± S.D. of triplicate experiments. Inset blot shows USP34-CD expression when overexpressed in HEK293T cells.

To identify additional negative regulators of TCR-mediated NF-κB activation, we conducted a siRNA library screen against 98 DUBs through a gene reporter luciferase assay in Jurkat T cells stimulated with either anti-CD3 and anti-CD28 antibodies or PMA plus ionomycin to mimic TCR engagement (Figure 1A and Additional files 1 and 2). As expected, CYLD silencing led to an enhanced NF-κB activity upon TCR stimulation (Figure 1A). Furthermore, this screening also uncovered siRNA sequences specific for USP34 that potentiated NF-κB activation with a similar magnitude to CYLD siRNA (Figure 1A). USP34 encompasses a 404 kDa protein with a central catalytic domain [11]. However, little is known about this DUB, albeit it was previously linked to the Wnt developmental signaling pathway [12]. Subcellular fractionation experiments showed that USP34 was essentially distributed in the cytosol of cells regardless of TCR stimulation, and was notably absent from the nucleus and organelles (Figure 1B and Additional file 3A). We next verified by immunoblot that CYLD and USP34 endogenous levels were efficiently decreased by their respective siRNA sequences (Figure 1C). Of note, an additional siRNA duplex specific for USP34 was also included to reinforce our initial findings (named sequence 3). Consistent with the primary screening, NF-κB reporter activity was similarly boosted upon TCR stimulation in USP34- and CYLD-silenced Jurkat when compared to control non-targeting siRNA transfected cells (Figure 1D and E). As a consequence, the levels of the NF-κB targets NFKBIA (IκBα), interleukin-2 (IL-2) and TNFα, as measured by RT-PCR were increased in USP34-knocked down cells (Figure 1F). Accordingly, downstream IL-2 secretion was enhanced in supernatants of USP34-silenced cells (Figure 1G). Finally, ectopic expression of a plasmid encoding for the catalytic domain of USP34 (USP34-CD [13]) markedly dampened TCR-mediated NF-κB activity (Figure 1H). Because USP34-CD is a large segment (383 amino acids), it is possible that in addition to the catalytic domain, it also comprises a domain required for the binding to its partners to regulate NF-κB in lymphocytes. Collectively, our data suggest that USP34 is a cytosolic protein, which functions as a negative regulator of NF-κB upon TCR engagement.

**Figure 2:**
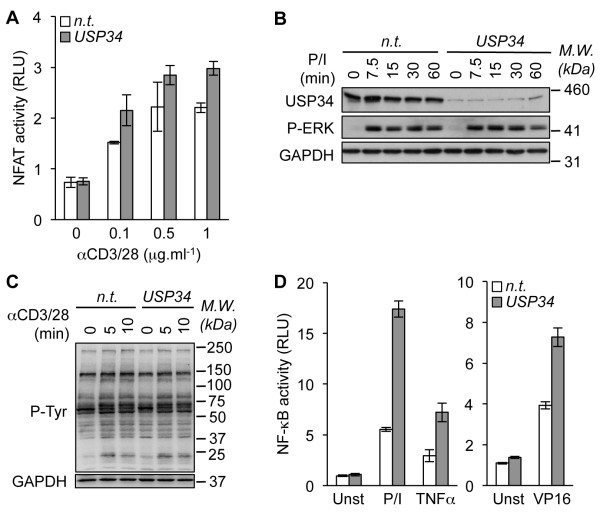
**Knockdown of USP34 selectively potentiates NF-κB activation.** (**A**) NFAT reporter luciferase assay (mean ± S.D. of triplicate experiments) of Jurkat cells transfected with control non-targeting (n.t.) or USP34 siRNA and stimulated with 0, 0.1, 0.5, and 1 μg.ml^-1^ anti-CD3 and anti-CD28 antibodies (αCD3/28). RLU, Relative Light Units. (**B**) n.t.- and USP34-silenced Jurkat cells were stimulated with 20 ng.ml^-1^ PMA plus 300 ng.ml^-1^ ionomycin (P/I) for 0, 7.5, 15, 30 and 60 min. Cell extracts were prepared and analyzed by immunoblot as indicated. Molecular weight markers (*M.W.*) are shown. (**C**) Cells as in (**B**) were stimulated with 1 μg.ml^-1^ anti-CD3 and anti-CD28 antibodies. Cell extracts were prepared and general tyrosine phosphorylation pattern (P-Tyr) was evaluated by immunoblot. GAPDH served as a loading control. (**D**) NF-κB reporter luciferase assay of Jurkat cells transfected as in (**A**) and stimulated with 10 ng.ml^-1^ PMA plus 300 ng.ml^-1^ ionomycin (P/I), with 10 ng.ml^-1^ TNFα, or with 40 μM etoposide (VP16). Shown is the mean ± S.D. of triplicate experiments. Unst, unstimulated.

In addition to NF-κB, TCR ligation kindles various signaling pathways including Nuclear factor of activated T-cells (NFAT) and the Mitogen-activated protein kinase (MAPK) Extracellular signal-regulated kinases (ERK) [14]. Gene reporter assays showed only modest increase in NFAT activation in USP34-silenced when compared to control cells (Figure 2A). Furthermore, ERK phosphorylation occurred normally without USP34 (Figure 2B). Keeping with this, no overt change in the general pattern of tyrosine phosphorylation was observed upon TCR stimulation, further arguing against a general impairment of TCR signaling in the absence of USP34 (Figure 2C). We next investigated whether USP34 also curtailed NF-κB activity emanating from TCR-autonomous signaling triggers. To this end, USP34-silenced Jurkat cells were stimulated with the cytokine TNFα or with the genotoxic stress agent etoposide that functions via an unconventional ATM/PIASy/Sumoylated-NEMO axis [15]. Paralleling the situation with TCR, knocking down USP34 markedly increased NF-κB in cells treated with TNFα or etoposide (Figure 2D). Supporting previous studies with CYLD-deficient cells [10,16], CYLD silencing in Jurkat cells also increased TNFα- and etoposide-mediated NF-κB activation (Additional file 4). Combined, these results indicate that USP34 shares some functional similarities with CYLD and selectively targets the NF-κB signaling pathway.

**Figure 3:**
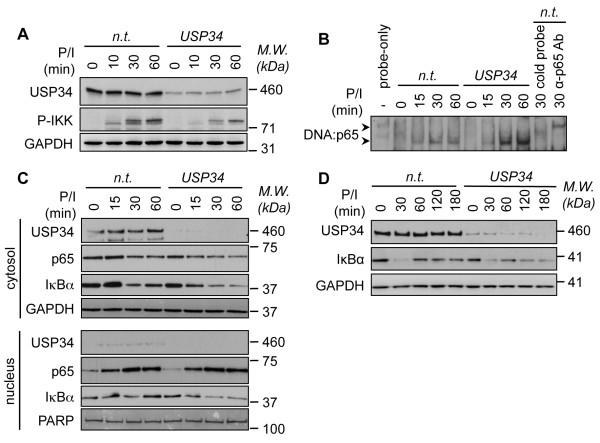
**Silencing of USP34 enhances IκBα degradation and NF-κB binding to DNA.** Jurkat cells were transfected with non-targeting- (n.t.) or USP34-targeting siRNA for 72-96 h. (**A**) Lysates from cells stimulated with 40 ng.ml^-1^ PMA plus 300 ng.ml^-1^ ionomycin (P/I) for 0, 10, 30 and 60 min were analyzed by immunoblot as indicated. *M.W.*, molecular weight markers. (**B**) NF-κB binding to DNA was assessed on nuclear extracts from cells stimulated as in (**A**) for the indicated time by non-radioactive electrophoretic mobility shift assays (EMSA) using biotin-labeled κB element DNA sequence. Note that DNA:p65 complexes were chased away with a cold probe, and were shifted when a p65 antibody was added. (**C**) Nuclear and cytoplasmic extracts from cells stimulated as in (**A**) were analyzed by immunoblot as indicated. GAPDH and PARP served as loading controls. (**D**) Lysates from cells stimulated as in (**A**) were assessed by immunoblot as indicated.

To gain insights on the signaling basis for the exacerbated NF-κB activity in USP34-depleted cells, we first examined BCL10 and MALT1 ubiquitinylation status since it governs the strength of TCR-mediated NF-κB activation [4-6]. BCL10 ubiquitinylation, which can be assessed in fractions enriched with heavy membranes [17], remained unchanged without USP34 (Additional file 3A). Moreover, pull-down of CK1α to precipitate the CBM complex [7,17], showed similar amounts of ubiquitinylated MALT1 bound in both control- and USP34-siRNA transfected cells (Additional file 3B). Keeping with this, BCL10 association to CARMA1 occurred normally without USP34 (Additional file 3C). We finally evaluated the impact of USP34 on the phosphorylation of IKK, which reflects its activation [8]. IKK phosphorylation was not exacerbated in those cells, and rather appeared slightly decreased (Figure 3A, and Additional file 3D). Although puzzling, this might result from a feedback loop triggered by enhanced NF-κB. We next assessed directly NF-κB DNA binding activity. Consistent with the gene reporter assays, more active NF-κB-DNA complexes were detected in nuclear extracts from TCR stimulated cells when USP34 was silenced (Figure 3B). Although no obvious difference in NF-κB subunit p65 translocation into the nucleus was detected, the degradation of the primary NF-κB inhibitor IκBα was more pronounced in cytosolic fractions from USP34-silenced cells when compared to control cells (Figure 3C). Accordingly, IκBα degradation was also prolonged or more dramatic in lysates from cells transfected with USP34 siRNA even on longer time points up to 3 hours (Figure 3D). Hence, our results suggest that USP34 likely functions downstream of the CBM-IKK nexus to enhance NF-κB activation.

Almost 100 DUBs were identified in the human genome and yet, only a few have been ascribed a function [11]. As for TCR signaling, the well studied A20 and CYLD thwart NF-κB at different levels [10]. CYLD targets and inhibits the ubiquitin-dependent IKKβ kinase TAK1 and therefore prevents aberrant lymphocyte activation [18,19], while A20 dampens NF-κB activity by trimming K63-ubiquitin chains attached to MALT1 [20,21]. Our study now unveils USP34 as an additional negative regulator of NF-κB in lymphocytes. How USP34 tempers NF-κB activity remains unclear. In contrast to CYLD and A20, which target apical signaling [18,20], USP34 rather seems to function downstream of the IKK complex. Given that USP34 does not bind to the NF-κB core components (our unpublished results), we favor a model in which USP34 impacts on the activity of a cytosolic co-activator to ensure IκBα fine-tuning [22,23]. Alternatively, USP34 might also intervene in other checkpoints to control NF-κB signal outcome and intensity such as post-translational modifications, nuclear shift, or DNA three-dimensional structure [22,24,25]. Nevertheless, our data illustrates how various layers of control cooperate to ensure the fine-tuning of NF-κB following engagement of the TCR.

## Abbreviations

CYLD: Cylindromatosis; DUBs: Deubiquitinylases; ERK: Extracellular signal-regulated kinases; IκBs: Inhibitors of NF-κB; IKK: IκBs kinase; MAPK: Mitogen-activated protein kinase; NFAT: Nuclear factor of activated T-cells; NF-κB: Nuclear factor-κB; PMA: phorbol 12-myristate 13-acetate; TCR: T-Cell receptor.

## Competing interests

The authors declare that they have no competing interests.

## Authors’ contributions

KP designed and conducted most experiments, analyzed the data and wrote the manuscript. EMH, NC, SMD, HML, CL, and CA designed and conducted experiments, and analyzed the data. EMH and JG helped drafting the manuscript. JG and AV analyzed the data. NB conceived the project, analyzed the data and wrote the manuscript. All authors read and approved the final version of the manuscript.

## Supplementary Material

Additional file 1 Methods description. Click here for file

Additional file 2 Design of the siRNA library screen. Click here for file

Additional file 3 BCL10 and MALT1 ubiquitinylation, CBM assembly, and IKK phosphorylation in USP34-silenced cells. Click here for file

Additional file 4 CYLD and USP34 silencing enhanced etoposide- and TNFα-mediated NF-κB activation. Click here for file
