# Supplementary material for: Negative regulation of NF-κB signaling in T lymphocytes by the ubiquitin-specific protease USP34
Source: Cell Commun Signal. 2013 Apr 16;11:25. doi: 10.1186/1478-811X-11-25 (PMC3649923; doi:10.1186/1478-811X-11-25)
Supplement: Additional file 1 — Methods description. [file 1478-811X-11-25-S1.pdf]

Additional File 1: Methods description

Cell culture and reagents

Jurkat T lymphocytes (clone E6.1) and human embryonic kidney 293T cells (HEK293T) were obtained from the ATCC. Cells were stimulated with 0.1-1 $\mu g.ml^{-1}$ of anti-CD3 and anti-CD28 antibodies (BD Biosciences), or with 10-40 $ng.ml^{-1}$ phorbol 12-myristate 13-acetate (PMA, Sigma) plus 300 $ng.ml^{-1}$ ionomycin (Calbiochem). TNFα (R&D) and etoposide (VP16, Sigma) were also used. Plasmid encoding the catalytic domain of USP34 was kindly provided by V. Quesada and C. Lopez-Otin [1], and was subcloned in a V5-backbone (Invitrogen, Life technologies).

siRNA library and transfections

siRNA library targeting 98 human DUBs (two oligoribonucleotide duplexes per target, based on [2]) was purchased from Sigma (see Additional File 2). Jurkat cells were transfected by electroporation with a BTX ECM 830 apparatus (BTX, Harvard Apparatus). siRNA sequences used were: USP34.1, 5'-GGAUCUAGCAAUGAGGUUA[dT][dT]-3' (Sigma); USP34.2, 5'-GAUCUUAGGGCUGAAGUAA[dT][dT]-3' (Sigma); USP34.3, 5'-GGCAAGACAUUUGGCUGACUGUAUU-3' (Invitrogen); CYLD, 5'-GAACAGAUUCCACUCUUUA[dT][dT]-3' (Sigma). Negative controls were from Sigma and Invitrogen.

Luciferase assays

Firefly luciferase constructs downstream of κB or NFAT-responsive elements were co-transfected with renilla luciferase pRL-TK (Int-) plasmid (Promega). Luciferase activities were analyzed by measuring light emission using the Dual-Luciferase Kit (Promega), with firefly

luminescence units normalized to renilla firefly luciferase luminescence units (BMG microplate reader).

Enzyme-linked ImmunoSorbent Assay (ELISA)

Human IL-2 production and release was determined after overnight stimulation, in the culture supernatants by an enzyme-linked immunosorbent assay (R&D).

Reverse Transcription-Polymerase Chain Reactions (RT-PCR)

RT-PCR were carried out as previously described [3]. Briefly, equal amounts of RNA (Qiagen RNeasy mini kit) were reverse transcribed (Superscript III, Invitrogen), and cDNA were then amplified (RedTaq ready mix, Sigma). Primers used were: NFKBIA, 5′-GCAAAATCCTGACCTGGTGT -3′ and 3′- GCTCGTCCTCTGTGAACTCC -5′; TNFα, 5’-TCCTTCAGACACCCTCAACC -3’ and 3’- AGGCCCCAGTTTGAATTCTT -5’; IL-2, 5’-ACCTCAACTCCTGCCACAAT -3' and 3’- GCCTTCTTGGGCATGTAAAA -5’; β-actin, 5′-AGCACTGTGTTGGCGTACAG -3′ and 3′- GGACTTCGAGCAAGAGATGG -5′.

Immunoblots and immunoprecipitation

Stimuli were washed out twice with PBS 1X and cell pellets were lysed at 4ºC with 50 mM Tris pH 7.4, 150 mM NaCl, 2 mM EDTA, 1% Triton X-100, 1% Igepal, supplemented with Complete protease inhibitors (Roche). 20 nM calyculin A was added to the lysis buffer when IKK phosphorylation was assessed. Lysates were cleared by centrifugation at 9,000g at 4ºC and total protein concentration was determinated with a micro BCA kit (Thermo scientific). 5-20 μg were resolved in 5-8% Tris-Acetate SDS-PAGE gels (Invitrogen) or 5-20% Tris-Glycine gels

according to company's instructions and electrotransfered onto nitrocellulose membranes (Amersham). Antibodies to USP34 were from Bethyl laboratories. Antibodies against BCL10 (A-6), CK1α (C-19), CYLD (H-6), LAMP2 (H4B4), MALT1 (B-12), NF-κB p65 (C-20 and F-6), and tubulin (TU-02) were from Santa Cruz Biotechnology. Antibodies against CARMA1 (1D12), IκBα, phosphorylated-IκBα (5A5), phosphorylated IKKαβ (16A6), and phosphorylated ERK1/2 (E10) were purchased from Cell Signaling Technology. Antibodies against GAPDH and V5 tag (Sigma), phosphorylated tyrosine (4G10, Millipore), and PARP (BD biosciences) were also used. HRP-conjugated secondary antibodies were purchased from Jackson. Immobilon chemiluminescent substrate (Millipore) was used for protein detection with autoradiography films. Immunoprecipitations were carried out as previously described [4]. Briefly, post-centrifugation lysates were precleared with protein G-sepharose (Roche) for 30 min prior to 1-2h incubation with antibodies and fresh protein G-sepharose at 4ºC. After four washes, immunocomplexes were denaturated and resolved by SDS-PAGE.

Electrophoretic Mobility Shift Assay (EMSA) and subcellular fractionation

$5\text{-}10.10^6$ cells were washed with PBS1X and placed for 5 min at 4ºC in 187.5 μl Buffer A (10 mM HEPES pH 7.4, 10 mM KCl, 0.1 mM EDTA, 0.1 mM EGTA, 1 mM DTT, 1 mM Na_3VO_4, 1 mM NaF, and Complete proteases inhibitors). Plasma membranes were solubilized by adding 12.5 μl of 10% Igepal (Sigma) for 5 min at 4ºC. Nuclei were pelleted with a 600*g* centrifugation and washed twice with buffer A. Nuclear proteins were then extracted with 30 μl Buffer C (20 mM HEPES pH 7.4, 400 mM NaCl, 1 mM EDTA, 1 mM EGTA, 1 mM DTT, 1 mM Na_3VO_4, 1 mM NaF, and Complete proteases inhibitors) under rocking conditions. Nuclear extracts were cleared by centrifugation at 13,000*g*. EMSA to detect NF-κB:DNA complex formation was

performed by using a non-radioactive kit with biotin-labeled or unlabeled DNA sequences (5'-AGTTGAGGGGACTTTCCCAGGC-3') containing κB element (Affymetrix). Jurkat nuclear extracts were treated according to manufacturers protocol. Biodyne B nylon membranes (Thermo scientific) were used for the electrotransfer. Subcellular fractionation experiments were performed as previously described [5]. In brief, cells were mechanically permeabilized with $27G^{1/2}$ syringe (Becton Dickinson) in 20 mM HEPES pH 7.9, 1.5 mM $MgCl_2$, 60 mM KCl, and protease inhibitors. Nuclei were eliminated by a 1,000*g* centrifugation, and supernatants were further spun at 10,000*g* to yield crude heavy membranes pellets. Cytosolic fractions were obtained after an additional 25,000*g* centrifugation.

Additional References

1. Quesada V, Diaz-Perales A, Gutierrez-Fernandez A, Garabaya C, Cal S, Lopez-Otin C: **Cloning and enzymatic analysis of 22 novel human ubiquitin-specific proteases**. *Biochem Biophys Res Commun* 2004, **314**(1):54-62.
2. Nijman SM, Luna-Vargas MP, Velds A, Brummelkamp TR, Dirac AM, Sixma TK, Bernards R: **A genomic and functional inventory of deubiquitinating enzymes**. *Cell* 2005, **123**(5):773-786.
3. Dwyer J, Hebda JK, Le Guelte A, Galan-Moya EM, Smith SS, Azzi S, Bidere N, Gavard J: **Glioblastoma cell-secreted interleukin-8 induces brain endothelial cell permeability via CXCR2**. *PLoS One* 2012, **7**(9):e45562.
4. Bidere N, Ngo VN, Lee J, Collins C, Zheng L, Wan F, Davis RE, Lenz G, Anderson DE, Arnoult D *et al*: **Casein kinase 1alpha governs antigen-receptor-induced NF-kappaB activation and human lymphoma cell survival**. *Nature* 2009, **458**(7234):92-96.
5. Carvalho G, Le Guelte A, Demian C, Vazquez A, Gavard J, Bidere N: **Interplay between BCL10, MALT1 and IkappaBalpha during T-cell-receptor-mediated NFkappaB activation**. *Journal of cell science* 2010, **123**(Pt 14):2375-2380.
