# Supplementary material for: Negative regulation of NF-κB signaling in T lymphocytes by the ubiquitin-specific protease USP34
Source: Cell Commun Signal. 2013 Apr 16;11:25. doi: 10.1186/1478-811X-11-25 (PMC3649923; doi:10.1186/1478-811X-11-25)
Supplement: Additional file 2 — Design of the siRNA library screen. [file 1478-811X-11-25-S2.pdf]

Additional File 2: Design of the siRNA library

Name	RefSeq ID	siRNA ID or sense siRNA
**BAP1**	NM_004656	SASI_Hs01_00105395
		SASI_Hs01_00105396
**UCH-L1**	NM_004181	SASI_Hs01_00178415
		SASI_Hs01_00178416
**UCH-L3**	NM_006002	SASI_Hs01_00200423
		SASI_Hs01_00200424
**UCH-L5, UCH37**	NM_015984	SASI_Hs01_00142742
		SASI_Hs01_00142743
**USP1**	NM_001017415	SASI_Hs01_00157121
		SASI_Hs01_00157122
**USP2**	NM_171997	SASI_Hs01_00174976
		SASI_Hs02_00367754
**USP3**	NM_006537	SASI_Hs01_00023593
		SASI_Hs01_00023595
**USP4, UNPH**	NM_199443	SASI_Hs01_00028215
		SASI_Hs01_00028216
**USP5**	NM_003481	SASI_Hs01_00184929
		SASI_Hs01_00184930
**USP6, TRE-2, TRE17**	NM_004505	SASI_Hs02_00337765
		SASI_Hs02_00337766
**USP7, HAUSP**	NM_003470	SASI_Hs01_00079539
		SASI_Hs01_00079540
**USP8, UBPY**	NM_005154	SASI_Hs02_00339089
		SASI_Hs01_00136039
**USP9X, FAF-X**	NM_001039590	SASI_Hs01_00031439
		SASI_Hs02_00308597
**USP9Y, FAF-Y**	NM_004654	SASI_Hs01_00081909
		SASI_Hs02_00338066
**USP10**	NM_005153	SASI_Hs01_00213007
		SASI_Hs01_00213008
**USP11**	NM_004651	SASI_Hs01_00148685
		SASI_Hs01_00148686
**USP12, UBH1**	NM_182488	SASI_Hs01_00167303
		SASI_Hs01_00167305
**USP13, ISOT-3**	NM_003940	SASI_Hs01_00108438
		SASI_Hs01_00108439
**USP14, ISOT**	NM_005151	SASI_Hs01_00016087
		SASI_Hs01_00016089
**USP15**	NM_006313	SASI_Hs01_00059894
		SASI_Hs01_00059895
**USP16, UBPM**	NM_001001992	SASI_Hs01_00121496
		SASI_Hs02_00341586
**USP17L3**	XM_001720764 1 s	CACGUUAACUUUACACACUdTdT
		CUAUCAUUGCGGUCUUUGUdTdT
		GCAAUAUCCUGAGUGCCUUdTdT
		GUUGUCACGACGGACAUUAdTdT
		GCAACAAACUUGCCAAGAAdTdT
		GGAAGAUGUCCAUGAAUUUdTdT
		GACAUUACUUCUCUUAUGUdTdT
		CUCAAGAAGGCCAGUGGUAdTdT
		CACCUUAGACCACUGGAAAdTdT
		GAGAUUCUCCGAUGUCGCAdTdT
**USP18, UBP43**	NM_017414	SASI_Hs01_00221412

		SASI_Hs01_00221413
**USP19**	NM_006677	SASI_Hs01_00130241<br>SASI_Hs01_00130242
**USP20, VDU1**	NM_001008563	SASI_Hs01_00099343<br>SASI_Hs01_00099344
**USP21, USP23**	NM_001014443	SASI_Hs01_00177787<br>SASI_Hs01_00177788
**USP22**	XM_042698	SASI_Hs01_00276309<br>SASI_Hs01_00276310
**USP24**	XM_934749	SASI_Hs01_00301651<br>SASI_Hs01_00301652
**USP25**	NM_013396	SASI_Hs02_00344762<br>SASI_Hs01_00191397
**USP26**	NM_031907	SASI_Hs01_00043149<br>SASI_Hs01_00043150
**USP28**	NM_020886	SASI_Hs01_00077918<br>SASI_Hs01_00077919
**USP29**	NM_020903	SASI_Hs01_00032719<br>SASI_Hs01_00032720
**USP30**	NM_032663	SASI_Hs01_00155679<br>SASI_Hs01_00155680
**USP31**	NM_020718	SASI_Hs01_00020641<br>SASI_Hs01_00020642
**USP32**	NM_032582	SASI_Hs01_00086230<br>SASI_Hs01_00086231
**USP33, VDU2**	NM_201626	SASI_Hs01_00142382<br>SASI_Hs01_00142383
**USP34**	NM_014709	SASI_Hs01_00089169<br>SASI_Hs02_00346129
**USP35**	XM_935137	SASI_Hs01_00291572<br>SASI_Hs01_00291573
**USP36**	NM_025090	SASI_Hs02_00357787<br>SASI_Hs01_00194136
**USP37**	NM_020935	SASI_Hs01_00018875<br>SASI_Hs02_00354323
**USP38**	NM_032557	SASI_Hs01_00189218<br>SASI_Hs02_00360065
**USP39, SAD1**	NM_006590	SASI_Hs01_00034674<br>SASI_Hs01_00034675
**USP40**	NM_018218	SASI_Hs01_00145783<br>SASI_Hs02_00351527
**USP41**	XM_036729	SASI_Hs02_00482814<br>SASI_Hs02_00482815
**USP42**	XM_932196	SASI_Hs01_00248233<br>SASI_Hs01_00248234
**USP43**	XM_934221	SASI_Hs01_00274671<br>SASI_Hs01_00274672
**USP44**	NM_032147	SASI_Hs02_00359381<br>SASI_Hs01_00075742
**USP45**	XM_931124	SASI_Hs01_00265784<br>SASI_Hs01_00265785
**USP46**	NM_022832	SASI_Hs01_00080807<br>SASI_Hs01_00080808
**USP47**	NM_017944	SASI_Hs01_00112225<br>SASI_Hs01_00112227
**USP48**	NM_032236	SASI_Hs01_00077498<br>SASI_Hs01_00077499
**USP49**	NM_018561	SASI_Hs01_00055585

		SASI_Hs02_00352112
**USP50**	NM_203494	SASI_Hs02_00373416<br>SASI_Hs02_00373417
**USP51**	NM_201286	SASI_Hs02_00372924<br>SASI_Hs02_00372925
**USP52**	NM_014871	SASI_Hs01_00133440<br>SASI_Hs01_00133442
**USP54**	NM_152586	SASI_Hs02_00366408<br>SASI_Hs02_00366409
**USPL1**	NM_005800	SASI_Hs02_00340306<br>SASI_Hs01_00128479
**CYLD**	NM_015247	SASI_Hs01_00177192<br>SASI_Hs01_00177191
**DUB-3**	NM_201402	SASI_Hs02_00372940<br>SASI_Hs02_00372941
**Ataxin-3**	NM_030660	SASI_Hs02_00358236<br>SASI_Hs02_00358237
**Ataxin-3-like**	XM_045705	SASI_Hs01_00259290<br>SASI_Hs01_00259291
**Josephin-1**	NM_014876	SASI_Hs02_00346483<br>SASI_Hs01_00072064
**Josephin-2**	NM_138334	SASI_Hs01_00128488<br>SASI_Hs01_00128489
**TAF1D**	NM_024116	SASI_Hs01_00119698<br>SASI_Hs01_00119700
**TNFAIP3, A20**	NM_006290	SASI_Hs01_00033556<br>SASI_Hs01_00033557
**ZA20D1, Cezanne-1**	NM_020205	SASI_Hs01_00089029<br>SASI_Hs01_00089030
**OTUD7i, Cezanne-2**	NM_130901	SASI_Hs01_00055782<br>SASI_Hs01_00055783
**OTUD4, HIN-1**	NM_017493	SASI_Hs02_00372731<br>SASI_Hs02_00372732
**OTUD6A, HIN-6**	NM_207320	SASI_Hs01_00018704<br>SASI_Hs01_00018705
**OTUD2, YOD1**	NM_018566	SASI_Hs01_00052848<br>SASI_Hs01_00052849
**OTUD6B**	NM_016023	SASI_Hs01_00222466<br>SASI_Hs02_00348712
**OTUD5**	NM_017602	SASI_Hs01_00052828<br>SASI_Hs01_00052829
**OTUB1, Otubain-1**	NM_017670	SASI_Hs02_00378146<br>SASI_Hs02_00378147
**OTUB2, Otubain-2**	NM_023112	SASI_Hs01_00010701<br>SASI_Hs01_00010702
**OTUD1**	XR_040065	SASI_Hs02_00515603<br>SASI_Hs02_00515604
**ZRANB1, TRABID**	NM_017580	SASI_Hs01_00155779<br>SASI_Hs02_00350242
**VCPIP1, VCIP135**	NM_025054	SASI_Hs02_00357704<br>SASI_Hs01_00165636
**STAMBP, AMSH**	NM_006463	SASI_Hs01_00051759<br>SASI_Hs01_00051760
**STAMBPL1, AMSH-like**	NM_020799	SASI_Hs01_00241436<br>SASI_Hs01_00241437
**BRCC3i, BRCC36, BRCC3**	NM_001018055	SASI_Hs02_00356685<br>SASI_Hs01_00193368
**COPS5, CSN5, JAB1, SGN5**	NM_006837	SASI_Hs02_00342404

		SASI_Hs01_00209042
**COPS6, CSN6, SGN6**	NM_006833	SASI_Hs02_00342398<br>SASI_Hs01_00117938
**EIF3S3, eIF-3-gamma**	NM_003756	SASI_Hs01_00072290<br>SASI_Hs01_00072291
**EIF3S5, eIF-epsilon**	NM_003754	SASI_Hs01_00246686<br>SASI_Hs02_00336223
**MYSM1**	XM_055481	SASI_Hs01_00255872<br>SASI_Hs01_00255873
**MPND**	NM_032868	SASI_Hs01_00162447<br>SASI_Hs01_00162448
**PSMD14, POH1, RPN11**	NM_005805	SASI_Hs02_00340316<br>SASI_Hs01_00024446
**PRPF8, PRP8**	NM_006445	SASI_Hs01_00015918<br>SASI_Hs01_00015919
**PSMD7, RPN8**	NM_002811	SASI_Hs02_00334491<br>SASI_Hs02_00334492
**USP17L5**	XM_001130437	SASI_Hs02_00387302<br>SASI_Hs02_00387303
**USP17L1P**	XR_040280	SASI_Hs02_00517496<br>SASI_Hs02_00517497
**IFP38**	NG_005632 s	CCGAUGACUUUGAGACCAUdTdT
**USP17L3**	XM_001720764	CACGUUAACUUUACACACUdTdT<br>CUAUCAUUGCGGUCUUUGUdTdT<br>GCAAUAUCCUGAGUGCCUUdTdT<br>GUUGUCACGACGGACAUUAdTdT<br>GCAACAAACUUGCCAAGAAdTdT<br>GGAAGAUGUCCAUGAAUUUdTdT<br>GACAUUACUUCUCUUAUGUdTdT<br>CUCAAGAAGGCCAGUGGUAdTdT<br>CACCUUAGACCACUGGAAAdTdT<br>GAGAUUCUCCGAUGUCGCAdTdT
**USP17L4**	XM_001720370	CACGUUAACUUUACACACUdTdT<br>CUAUCAUUGCGGUCUUUGUdTdT<br>GCAAUAUCCUGAGUGCCUUdTdT<br>GUUGUCACGACGGAUAUUAdTdT<br>GCAACAAACUUGCCAAGAAdTdT<br>CACCUUAGACCACUGGAAAdTdT<br>GGAAGAUGUCCAUGAAUUUdTdT<br>CUCAAGAAGGCCAGUGGUAdTdT<br>GAGAUUCUCCGAUGUCGCAdTdT<br>CGGAUAUUACUUCUCUUAUdTdT
**USP17L6**	NR_027279	CUGGACAUCGCCCUGGAUAdTdT<br>CCAAGGUCCUCAUCCUUGUdTdT<br>GAGAUUCUCCGAUGUCACAdTdT<br>GCCUAUCAUUGUGGUGUUUdTdT<br>GCUUCCUUGCAGUGCCUGAdTdT<br>GGAAGAUGCCCAUGAAUUUdTdT
**USP17L7**	XM_001723172	GAGAUUCUCCGAUGUCACAdTdT<br>GCGUAUUGGAGAUCUCAAAdTdT<br>CACCUUAGACCACUGGAAAdTdT<br>CUCUCAAACGUGUCAUCUUdTdT<br>CUCAUUCUUGUAUUGAAGAdTdT<br>CCAAAGAACUCAAUGGAGAdTdT<br>GACGUUAACUUUACCCACUdTdT<br>CAUGAAUUUCUCAUGUUUAdTdT<br>CAUGUUCUGUACUAUGCAAdTdT<br>CUCAAGAAGGCCAGUGGUAdTdT

**USP17L8**	XM_001720762	GAGAUUCUGCGAUGUCACAdTdT<br>GUUGUCACAACGGAUAUUAdTdT<br>CUAUCCUUGCGGUCUUUGUdTdT
**DUSP19**	AB038770	GAAUUUAUUGAAGAAGCAAdTdT<br>GUUGCAUAUGGAGUUGAAAdTdT<br>CCAACAUCCUGUCUUAUUUdTdT<br>GACUUUACAUAUAAGAGCAdTdT<br>GCUUUCCUCAGUGACUUUAdTdT<br>GACUCAUAUUCUUAAUGUUdTdT
