# Supplementary figures and images for: Negative regulation of NF-κB signaling in T lymphocytes by the ubiquitin-specific protease USP34
Source: Cell Commun Signal. 2013 Apr 16;11:25. doi: 10.1186/1478-811X-11-25 (PMC3649923; doi:10.1186/1478-811X-11-25)

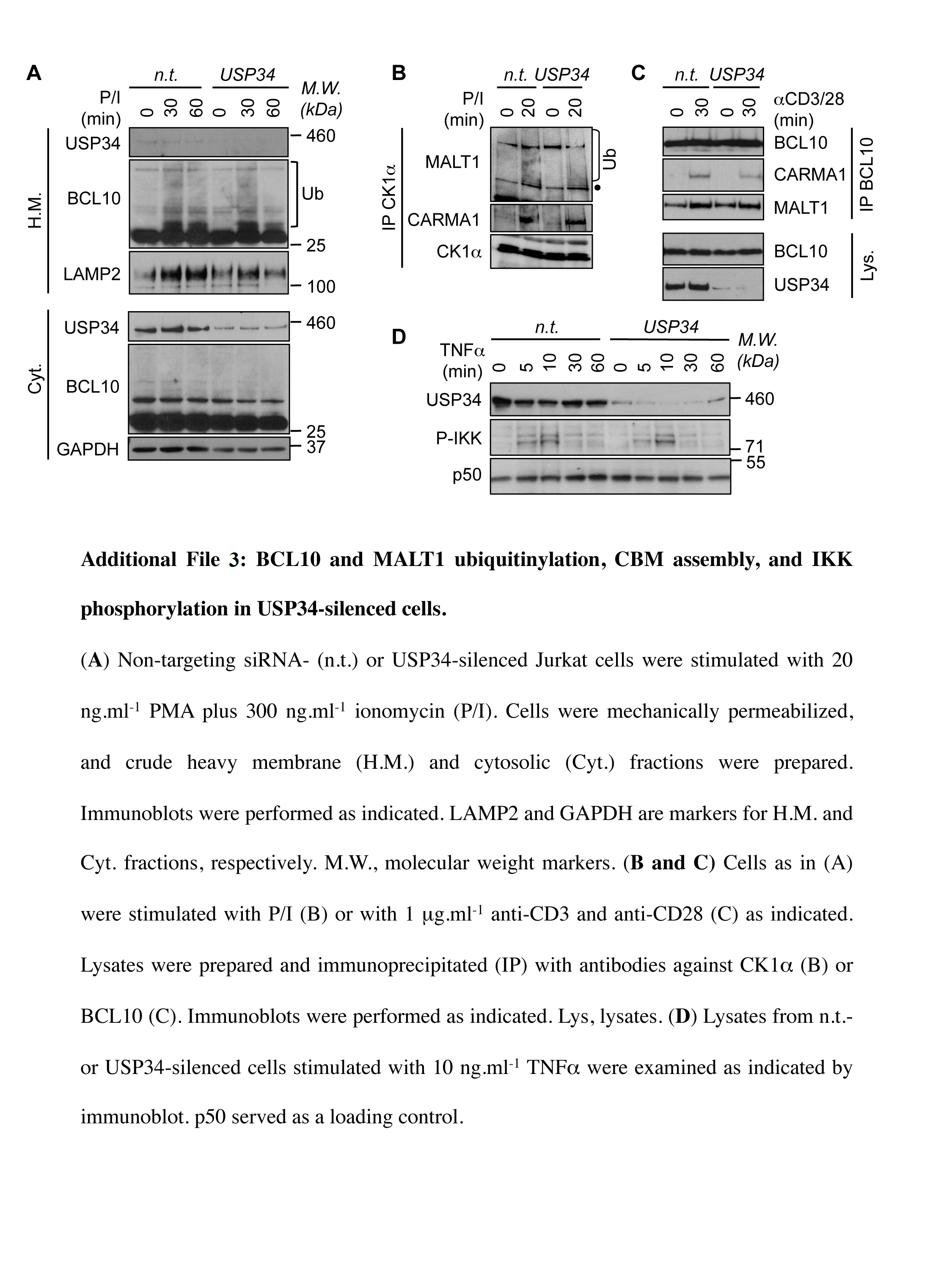

Supplement: Additional file 3 — BCL10 and MALT1 ubiquitinylation, CBM assembly, and IKK phosphorylation in USP34-silenced cells. [file 1478-811X-11-25-S3.tiff]

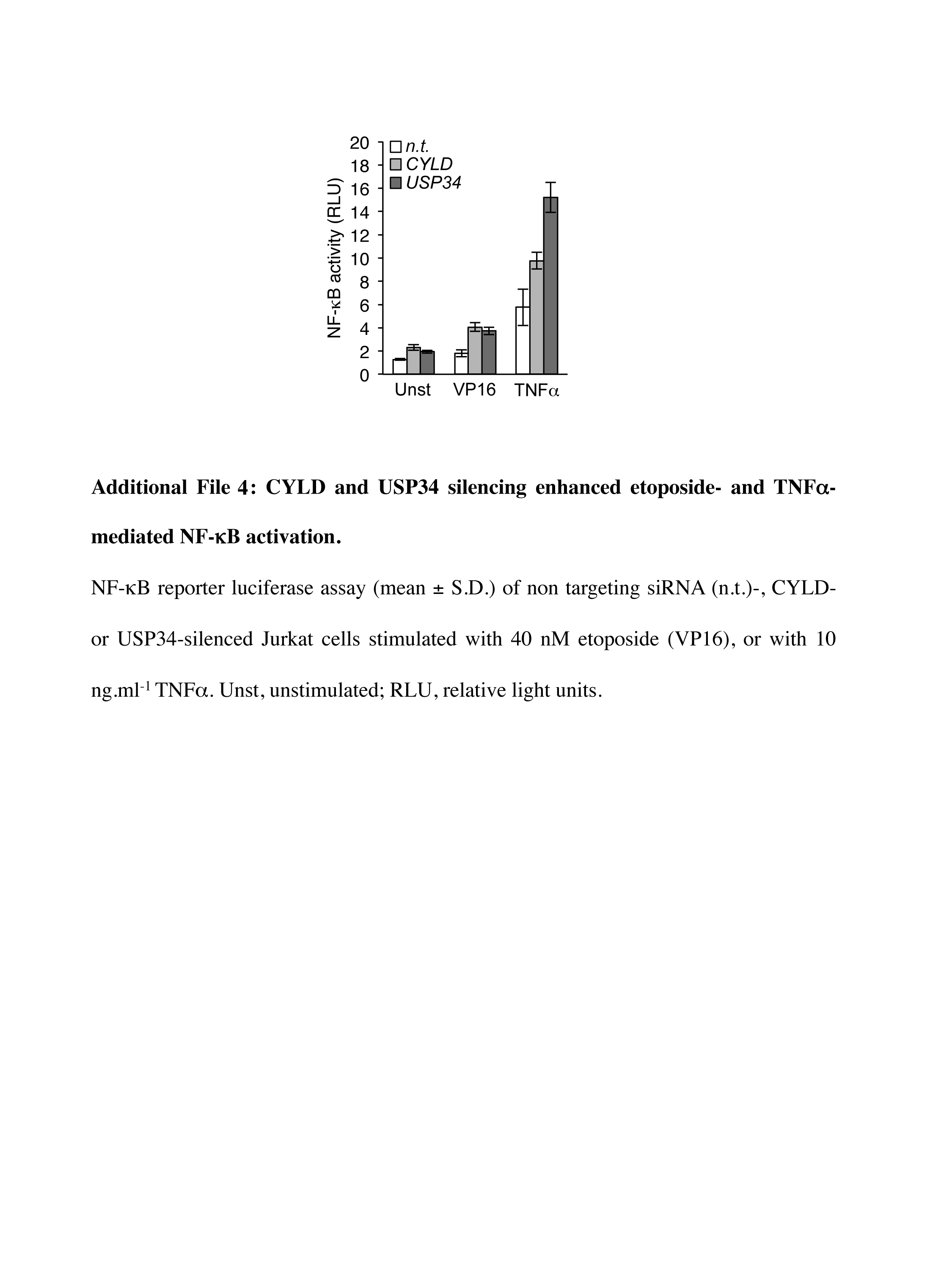

Supplement: Additional file 4 — CYLD and USP34 silencing enhanced etoposide- and TNFα-mediated NF-κB activation. [file 1478-811X-11-25-S4.tiff]
